# Effect of mind-body exercise on risk factors for metabolic syndrome including insulin resistance: a meta-analysis

**DOI:** 10.3389/fendo.2024.1289254

**Published:** 2024-01-26

**Authors:** Shufan Li, Peng Wang, Jing Wang, Jinlei Zhao, Xing Wang, Tong Liu

**Affiliations:** ^1^ School of Physical Education, Shanghai University of Sport, Shanghai, China; ^2^ School of Physical Education and Health, Shanghai Lixin University of Accounting and Finance, Shanghai, China; ^3^ Physical Education Department of Shanghai Industry and Commerce Foreign Languages College, Shanghai, China

**Keywords:** mind-body exercise, metabolic syndrome, insulin resistance, meta-analysis, randomized controlled trials

## Abstract

**Objective:**

To systematically evaluate the effects of mind-body exercise on risk factors of metabolic syndrome such as insulin resistance.

**Methods:**

Web of Science, PubMed, The Cochrane Library, EBSCO host, Embase, China Knowledge Network, China Biomedical Literature Database, Wanfang, and VIP were searched for the period from the establishment of the database to 1 July 2023, and randomized controlled trials of mind-body exercise interventions in patients with metabolic syndrome were collected. We applied the Cochrane Risk of Bias tool RoB2 to evaluate the methodological quality of the included literature and used RevMan5.4 software and Stata15.1 for statistical analysis.

**Results:**

A total of 14 randomized controlled trials with 1148 patients were included in this study. Meta-analysis showed that mind-body exercise significantly improved insulin resistance [SMD=-0.78, 95% CI: (-1.13, -0.43), *P*<0.0001], waist circumference [SMD=-2.20, 95% CI: (-3.34, -1.06), *P*=0.0001], body mass index (SMD=-1.50, 95% CI: [-2.03, -0.97), *P*<0.00001], systolic blood pressure [SMD=-3.65, 95% CI: 9-5.56, -1.74), *P*=0.0002], diastolic blood pressure [SMD=-3.32, 95% CI: (- 3.77, -2.87), *P*<0.00001], fasting blood glucose [SMD=-0.57, 95% CI: (-0.99, -0.15), *P*=0.008], triglycerides [SMD=-0.27, 95% CI: (-0.46, -0.08), *P*=0.004], high-density lipoprotein cholesterol [SMD=0.58, the 95% CI: (0.28, 0.87), *P*=0.0001]. Subgroup analysis showed that the intervention program with exercise form of fitness qigong, exercise cycle of 24-48 weeks, and exercise frequency of 6-7 times/week could significantly improve each risk factor.

**Conclusion:**

Mind-body exercise is effective in improving risk factors in patients with metabolic syndrome. Current evidence recommends an intervention program of low to moderate intensity fitness qigong, with 6-7 sessions per week for 24-48 weeks in patients with metabolic syndrome.

**Systematic review registration:**

https://www.crd.york.ac.uk/prospero/, identifier CRD42023454135.

## 1 Introduction

Metabolic syndrome (MetS) refers to a pathological condition in which various metabolic components in the body are abnormally aggregated, including central obesity, hypertension, glucose or insulin metabolism disorders, and lipid abnormalities. The pathological mechanisms involve insulin resistance and obesity (1, 2). MetS is a significant risk factor for cardiovascular diseases, type 2 diabetes, stroke, and other conditions (3, 4). It is also an important clinical indicator for predicting overall mortality and cardiovascular mortality (5). The prevalence of MetS is on the rise globally, with the prevalence in Chinese adults increasing from 8.8% in 1991 to 29.3% in 2015 (6). In the United States, the prevalence among adults is 35%, reaching close to 50% in the population aged 60 and above (7).

Currently, preventive and therapeutic measures for MetS primarily involve non-pharmacological and pharmacological interventions. Non-pharmacological treatments mainly focus on dietary control and exercise intervention, while pharmacological treatments target blood sugar, blood lipids, blood pressure, and other related aspects. Exercise is a crucial component of health management for individuals with MetS, offering the advantages of simplicity, feasibility, and minimal side effects (8). Moderate-intensity exercise has been shown to effectively reduce the occurrence of MetS (9–11). Mind-body exercise refers to activities that simultaneously engage both the body and consciousness. The core of this exercise involves training individuals to consciously control the activities of various parts of their bodies, achieving a harmonious unity of body and mind (12). Mind-body exercises are characterized by gentle and slow movements (moderate to low-intensity exercise), coordinated body movements with breathing, and common examples include yoga, Tai Chi, and fitness qigong (12, 13). Existing research has found that mind-body exercise can improve specific risk factors for MetS, such as obesity, hypertension, hyperglycemia, and dyslipidemia (14–17). Regular mind-body exercise can reduce body fat, enhance insulin sensitivity and reactivity in peripheral target tissues through skeletal muscle exercise, increase the utilization of glucose, alleviate insulin resistance, and improve skeletal muscle blood flow, promoting glucose uptake and metabolism in skeletal muscles, thereby ameliorating metabolic syndrome (18).

Different mind-body exercises have varying intervention effects. Yoga has a significant impact on waist circumference (WC) and systolic blood pressure (SBP) in individuals with MetS (19). Tai Chi can improve obesity, blood pressure, blood sugar, and blood lipids in community-dwelling adults at risk for MetS (15). Fitness qigong can improve WC, blood pressure, and lipid abnormalities in MetS patients (17). By reviewing previous research, it is noted that the forms of mind-body exercises included in previous meta-analyses were limited, outcome indicators were restricted, insulin resistance was not analyzed, and the impact of various elements of exercise on outcome indicators was not investigated. To address these gaps, this study aims to answer the following questions: Can mind-body exercise effectively improve insulin resistance and obesity in MetS patients? Can it effectively regulate blood pressure, blood sugar, and blood lipids in MetS patients? Which type of mind-body exercise intervention is more suitable for MetS patients? This study will comprehensively collect published randomized controlled trials, systematically evaluate the intervention effects of mind-body exercise on insulin resistance and other risk factors for metabolic syndrome, and provide scientific exercise programs for the prevention and improvement of metabolic syndrome, offering reliable evidence for clinical practice.

## 2 Research methods

This study followed the international guidelines for writing Meta-analyses (20) for the selection and use of research methods, PROSPERO registration number: CRD42023454135.

### 2.1 Literature search strategies

Two researchers conducted searches across nine databases, including Web of Science, PubMed, The Cochrane Library, EBSCO host, Embase, China Knowledge Network, China Biomedical Literature Database, Wanfang, and VIP. and supplemented with literature retrospectives, all of which were searched for the period of construction through July 1, 2023. The English search utilized the following subject terms and their free word combinations: Exercise, physical activity, movement, Mind-Body Exercise, Yoga, Traditional sports, Tai chi, Baduanjin, Wuqinxi, Yijinjing, Liuzijue, Qigong, Metabolic Syndrome, Insulin Resistance Syndrome X, randomized controlled trial.

### 2.2 Literature inclusion criteria

(1) The study subjects were patients diagnosed with metabolic syndrome by authoritative criteria. (2) The interventions were mind-body exercises, including yoga, tai chi, and fitness qigong (Baduanjin, Wuqinxi, and Yijinjing); and the control group could be treated with usual care, health promotion, etc. (3) The main outcome indicators were insulin resistance and obesity. Insulin resistance was selected as insulin resistance index (HoMA-IR), and waist circumference (WC) and body mass index (BMI) were selected for obesity. Secondary outcome indicators were blood pressure, blood glucose, and blood lipids. Systolic blood pressure (SBP) and diastolic blood pressure (DBP) were selected for blood pressure, fasting blood glucose (FBG) for blood glucose, and triglycerides (TG) and high-density lipoprotein cholesterol (HDL-C) for blood lipids. (4) The type of literature included was randomized controlled trial (RCT).

### 2.3 Literature exclusion criteria

(1) Animal experiments. (2) Review and conference literature. (3) Combined intervention studies with interventions other than exercise. (4) Literature data could not be extracted.

### 2.4 Data extraction

Literature inclusion and exclusion were strictly based on predefined criteria. Two individuals independently screened the literature, cross-checked the screened literature, and any discrepancies were resolved through discussion and evaluation by a third person. The screened literature was subjected to data extraction, cross-checking and if there were discrepancies in the data, the data was verified by a third person. Data extracted included: type of study, age, diagnostic criteria, sample size, intervention method and type of study. Data extracted included:(1) basic information (first author, year of publication, country, age, diagnostic criteria); (2) experimental information (sample size, intervention, exercise variables); (3) outcome indicators.

### 2.5 Quality assessment

Adopting the Cochrane Handbook of Systematic Evaluation 6.3 criteria for evaluating risk of bias, the Revised Cochrane risk-of-bias tool for randomized trials (RoB2) was used to assess the risk of bias in 5 areas. The methodological quality of the inclusion literature was evaluated independently by 2 researchers, and if there were disagreements, they were resolved by a third person in a joint discussion.

### 2.6 Data processing

Data extracted from the literature were subjected to systematic Meta-analysis in Review Manager 5.4 software. Measures with the same measurement tool were expressed as weighted mean difference (MD) and its 95% confidence interval (CI), while measures with different measurement tools were expressed as standardized mean difference (SMD) and its 95% CI. The supplement and clarification have been added to the data processing. In this study, Cochran’s Q test was employed, along with the I^2^ statistic, to assess the magnitude of heterogeneity. When *P* > 0.1 and I^2^ < 50%, it was considered that there was small heterogeneity among the studies, and a fixed-effects model was used to combine the effect size (the fixed-effects model assumes that all included studies share a common true effect size, or that, apart from random error, the observed effect sizes are all true effect sizes). When *P* < 0.1 or I^2^ > 50%, it was considered that there was significant heterogeneity among the studies, and a random-effects model was used to combine the effect size (the random-effects model assumes that the true effect size varies with the inclusion of different studies). Publication bias test was performed using the Begg test of Stata15.1 software.

## 3 Results

### 3.1 Literature screening results

**Figure 1:**
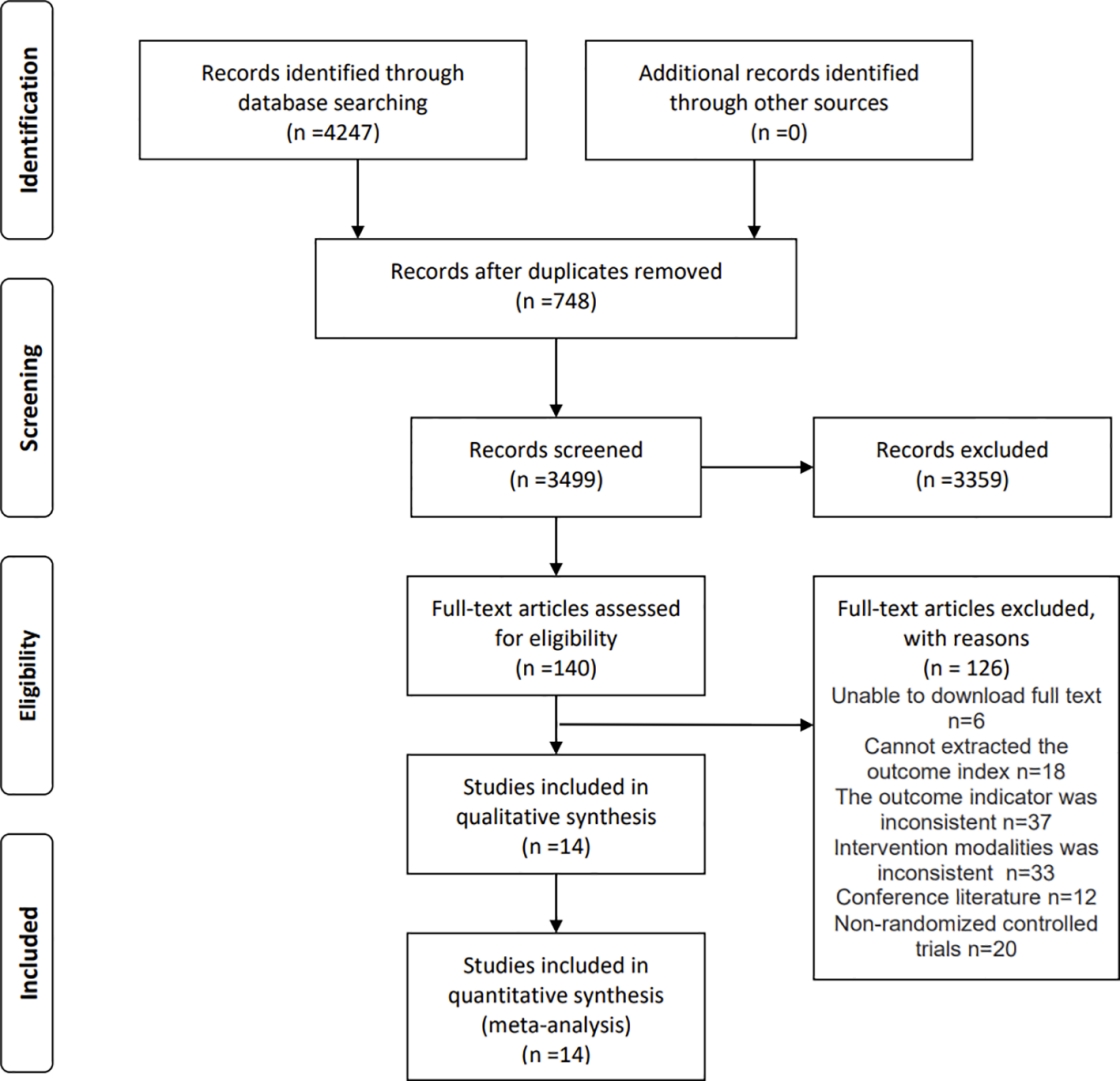
Flow Diagram of Literature Search.

The computer searched yielded 4247 documents, and 3499 documents were obtained after removing duplicates; 3359 documents were excluded after initial screening by reading the titles and abstracts, and 140 documents were left; after further full-text re-screening, 126 documents that did not meet the inclusion criteria were excluded, and finally 14 documents were included in the meta-analysis. see **Figure 1** for details.

### 3.2 Basic characteristics of the included literature

**Table 1** presents the basic information of the 14 included RCTs, involving a total of 1,148 participants aged between 40 and 73. Interventions included yoga, tai chi, baduanjin, wuqinxi, and yijinjing, conducted over periods ranging from 10 to 48 weeks, with 2 to 7 sessions per week and durations between 30 and 90 minutes.

**Table 1:** Basic Characteristics of the Literature.

Study	Country	Sample size(T/C)	Diagnostic criteria	Age (years) T/C	Intervention T/C	Duration (week)	Frequency (times/week)	Intervention time (min)	Measurements
Cohen 2008 (21)	United States	12/12	NCEP	52 ± 8/ 52 ± 9	yoga/usual care	10	4-5	30-90	WC, BMI, SBP, DBP, FBG, TG, HDL-C
Kanaya 2014 (22)	United States	72/63	IDF	55 ± 7/ 54 ± 7	yoga/ Stretching	48	4-5	30-90	HoMA-IR, WC, SBP, FBG, TG, HDL-C
Kim 2013 (23)	Korea	17/20	NCEP	48.2 ± 7.21/ 50.3 ± 8.30	yoga/usual care	12	2	60	WC, SBP, DBP, FBG, TG
Leung 2019 (24)	China	18/17	NCEP	62.19 ± 5.93/ 65.52 ± 9.34	Tai chi/ usual care	12	5	30-60	WC, SBP, DBP, FBG, TG
Mendoza-Núñez 2018 (25)	Mexico	48/37	NCEP	67.4 ± 4.7/ 68.2 ± 6.6	Tai chi/ usual care	24	5	50	BMI, SBP, DBP, FBG
Siu 2015 (26)	China	84/98	NCEP	56.3 ± 8.8/ 55.7 ± 9.4	yoga/usual care	48	3	60	WC, SBP, DBP, FBG, TG
Liu 2012 (27)	China	22/18	A	68.17 ± 3.50/ 67.9 ± 5.00	Wuqinxi/ usual care	24	**7**	60	WC, BMI, SBP, DBP, FBG, TG
Sun 2019 (28)	China	15/15	IDF	40-50	Wuqinxi/ usual care	24	5	60	HoMA-IR, FBG, TG
Sun 2015 (29)	China	15/15	IDF	40-50	Wuqinxi/ usual care	24	**7**	60	HoMA-IR, BMI, SBP, DBP, FBG, TG
Liao 2013 (30)	China	70/70	IDF	60.5 ± 11.8/ 62.7± 9.5	Baduanjin/ usual care	24	7	30	WC, BMI, SBP, DBP, FBG, TG
Yang 2023 (31)	China	41/39	IDF	72.71 ± 4.89/ 70.9 ± 4.27	Baduanjin/ usual care	24	7	60	WC, BMI, SBP, DBP, FBG
Zou 2013 (32)	China	100/100	IDF	57.42 ± 6.67/ 57.52 ± 6.2	Yijinjing/ usual care	24	7	30	BMI, FBG, TG
Jin 2021 (33)	China	45/45	B	69.7 ± 11.2/ 69.7 ± 11.2	Baduanjin/ usual care	12	7	30	SBP, DBP, FBG, TG
Wei 2017 (34)	China	20/20	IDF	45.2 ± 4.4	Baduanjin/ usual care	24	6	45	BMI, SBP, DBP, FBG, TG

T/C, experimental group/control group; min, minues; NCEP, National Cholesterol Education Program metabolic syndrome criteria; IDF, International Diabetes Federation metabolic syndrome criteria; A, diagnostic criteria recommended by the Diabetes Branch of the Chinese Medical Association in 2004; B, diagnostic criteria of the Guidelines for the Prevention and Control of Type 2 Diabetes Mellitus in China formulated by the Diabetes Branch of the Chinese Medical Association (2017 Edition) diagnostic criteria by the Chinese Medical Association Diabetes Branch; HoMA-IR, insulin resistance index; WC, waist circumference; BMI, body mass index; SBP, systolic blood pressure; DBP, diastolic blood pressure; FBG, fasting blood glucose; TG, triglycerides; HDL-C, high-density lipoprotein cholesterol.

### 3.3 Methodological quality assessment of the included literature

**Figure 2:**
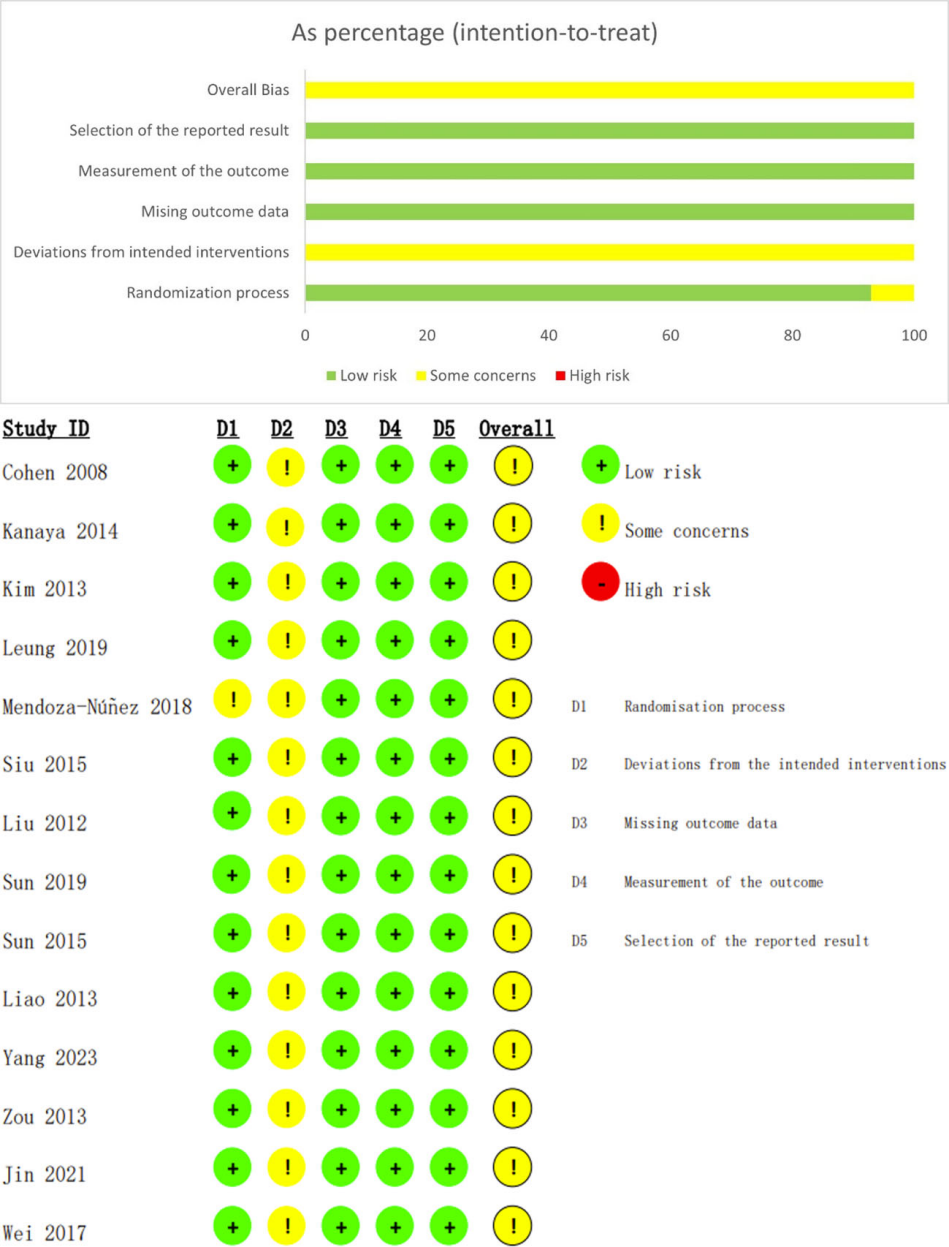
Risk of bias assessment of included studies.

As shown in **Figure 2**, a total of 14 randomized controlled trials were included. One study (25) had unclear bias in the randomization process, and all studies were rated as “Some concerns” for bias in the implementation of the predefined interventions. No studies showed bias related to outcome data missing, outcome measurement bias, or selective reporting bias.

### 3.4 Meta-analysis results

#### 3.4.1 Effects of mind-body exercise on insulin resistance

**Figure 3:**
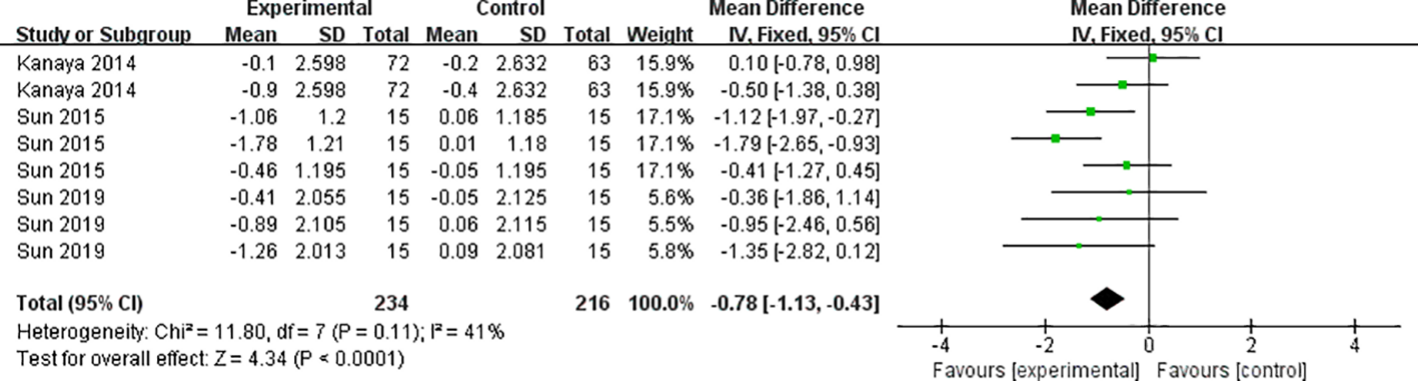
Meta-analysis of insulin resistance.

The three included papers (comprising 8 studies) reported the results of the Insulin Resistance Index (**Figure 3**), with low heterogeneity between studies (I²=41%, *P*=0.11), and they were analyzed using a fixed-effects model. Mind-body exercise reduced insulin resistance in patients with metabolic syndrome [SMD=-0.78, 95% CI: (-1.13, -0.43), *P*<0.0001], and the difference was statistically significant compared to the control group.

#### 3.4.2 Effects of mind-body exercise on obesity

##### 3.4.2.1 Waist circumference

**Figure 4:**
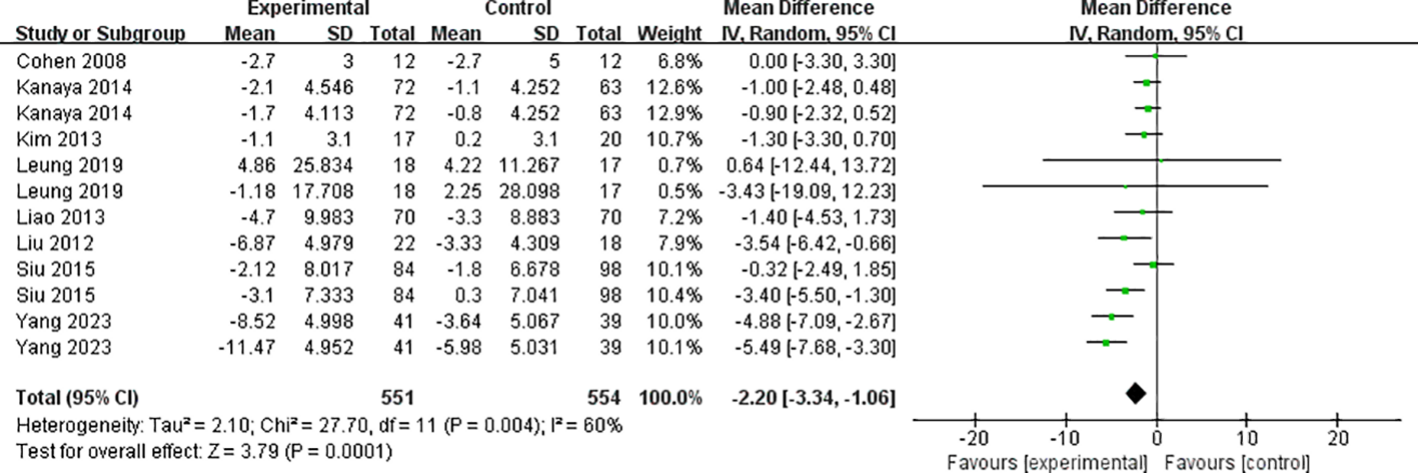
Meta-analysis of waist circumference.

The eight included papers (comprising 12 studies) reported the results of waist circumference (**Figure 4**), with high heterogeneity between studies (I²=60%, *P*=0.004), and they were analyzed using a random-effects model. Mind-body exercise reduced waist circumference in patients with metabolic syndrome [SMD=-2.20, 95% CI: (-3.34, -1.06), *P*=0.0001], and the difference was statistically significant compared to the control group.

##### 3.4.2.2 Body mass index

**Figure 5:**
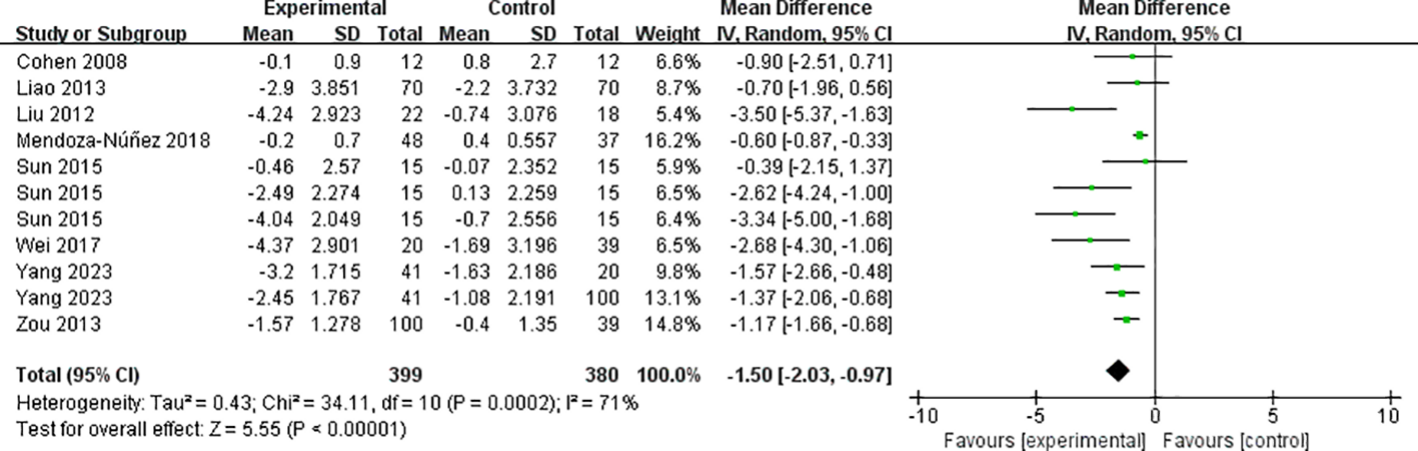
Meta-analysis of body mass index.

The eight included papers (consisting of 11 studies) reported the results of body mass index (**Figure 5**), with high heterogeneity between studies (I² = 71%, P = 0.0002), and they were analyzed using a random-effects model. Mind-body exercise reduced body mass index in patients with metabolic syndrome [SMD = -1.50, 95% CI: (-2.03, -0.97), P < 0.00001], and the difference was statistically significant compared to the control group.

#### 3.4.3 Effect of mind-body exercise on blood pressure

##### 3.4.3.1 Systolic blood pressure

**Figure 6:**
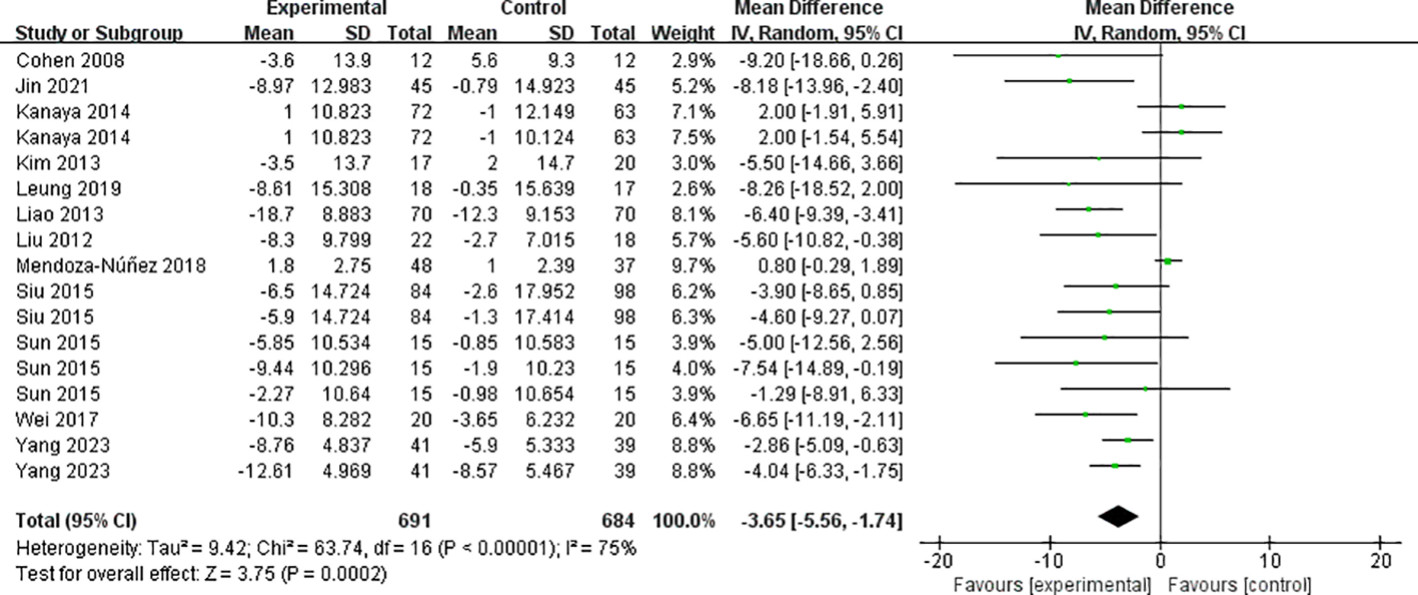
Meta-analysis of systolic blood pressure.

The twelve included papers (comprising 17 studies) reported the results of systolic blood pressure (**Figure 6**), with high heterogeneity between studies (I² = 75%, *P* < 0.00001), and they were analyzed using a random-effects model. Mind-body exercise reduced systolic blood pressure in patients with metabolic syndrome [SMD = -3.65, 95% CI: (-5.56, -1.74), *P* = 0.0002], and the difference was statistically significant compared to the control group.

##### 3.4.3.2 Diastolic blood pressure

**Figure 7:**
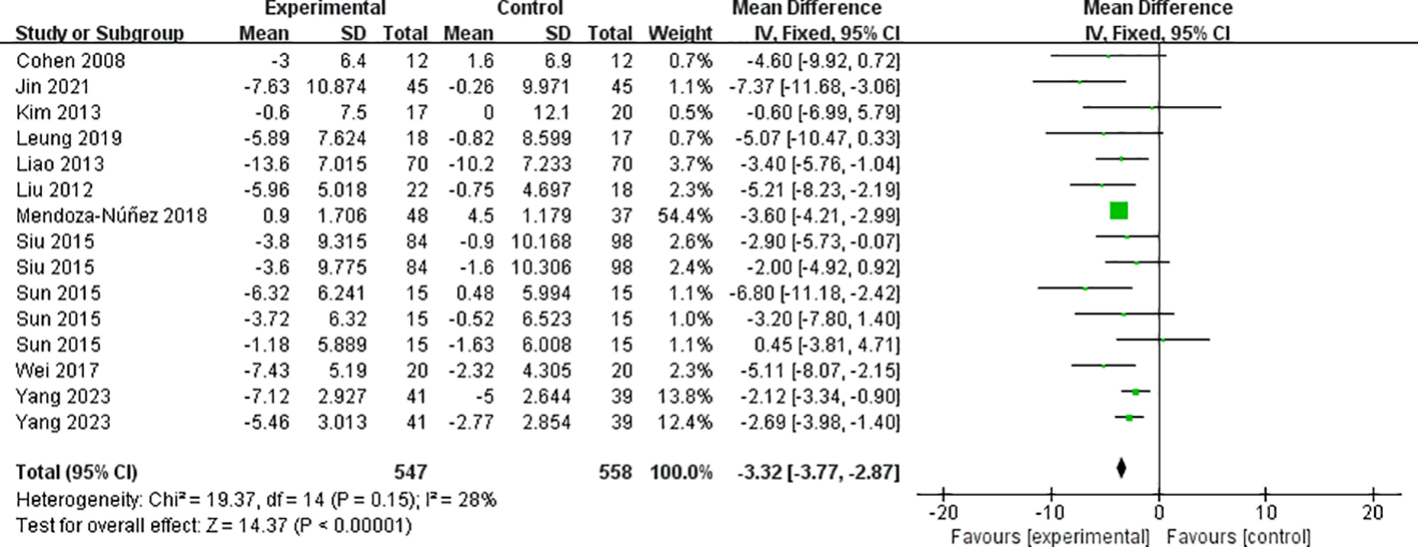
Meta-analysis of diastolic blood pressure.

The eleven included papers (comprising 15 studies) reported the results of diastolic blood pressure (**Figure 7**), with low heterogeneity between studies (I² = 28%, *P* = 0.15), and they were analyzed using a fixed-effects model. Mind-body exercise reduced diastolic blood pressure in patients with metabolic syndrome SMD = -3.32, 95% CI: (-3.77, -2.87), *P* < 0.00001], and the difference was statistically significant compared to the control group.

#### 3.4.4 Effect of mind-body exercise on blood glucose

**Figure 8:**
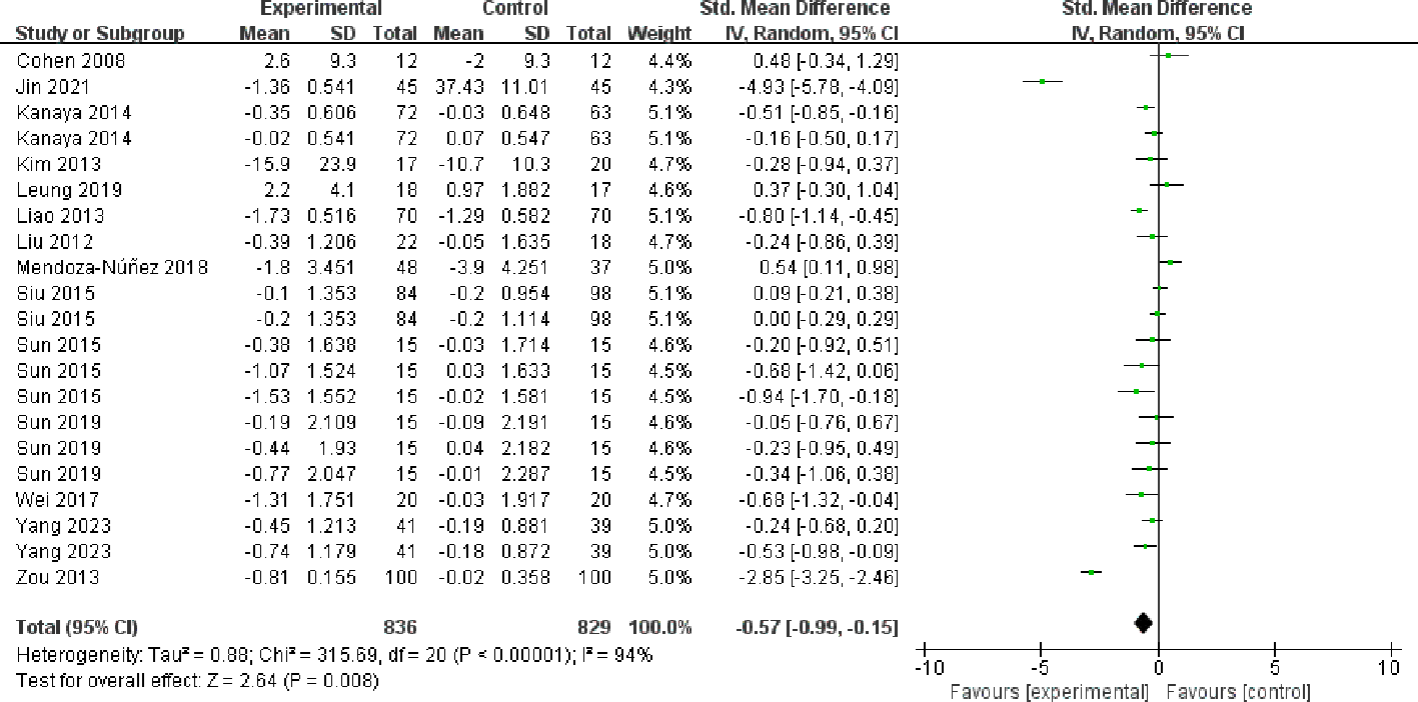
Meta-analysis of fasting blood glucose.

All 14 included papers (comprising 21 studies) reported the results of fasting blood glucose (**Figure 8**), with high heterogeneity between studies (I² = 94%, *P* < 0.00001), and they were analyzed using a random-effects model. Mind-body exercise reduced fasting blood glucose in patients with metabolic syndrome [SMD = -0.57, 95% CI: (-0.99, -0.15), *P* = 0.008], and the difference was statistically significant compared to the control group.

#### 3.4.5 Effects of mind-body exercise on blood lipids

##### 3.4.5.1 Triglycerides

**Figure 9:**
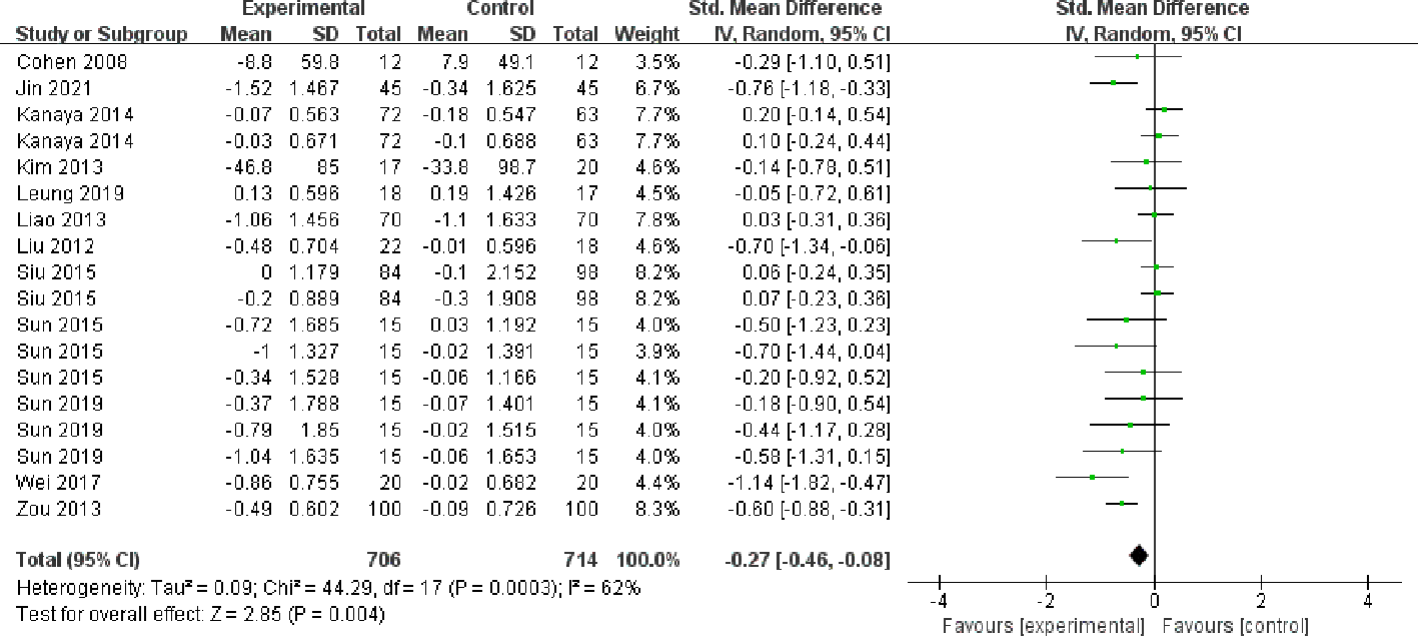
Meta-analysis of triglycerides.

The twelve included papers (comprising 18 studies) reported the results of triglycerides (**Figure 9**), with high heterogeneity between studies (I² = 62%, *P* = 0.0003), and they were analyzed using a random-effects model. Mind-body exercise reduced triglycerides in patients with metabolic syndrome [SMD = -0.27, 95% CI: (-0.46, -0.08), *P* = 0.004], and the difference was statistically significant compared to the control group.

##### 3.4.5.2 High-density lipoprotein cholesterol

**Figure 10:**
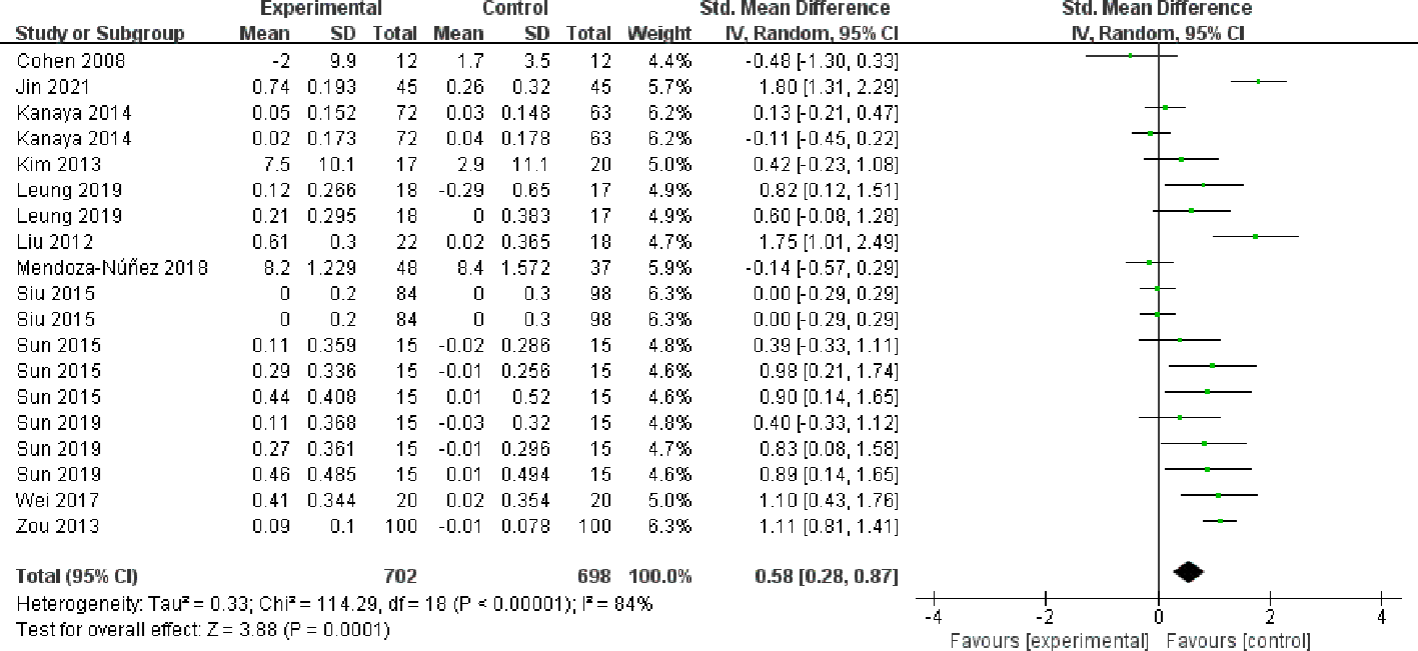
Meta-analysis of High-density lipoprotein cholesterol.

The twelve included papers (comprising 19 studies) reported the results of High-density lipoprotein cholesterol (**Figure 10**), with high heterogeneity between studies (I² = 84%, *P* < 0.00001), and they were analyzed using a random-effects model. Mind-body exercise could improve High-density lipoprotein cholesterol in patients with metabolic syndrome [SMD = 0.58, 95% CI: (0.28, 0.87), *P* = 0.0001], and the difference was statistically significant compared to the control group.

#### 3.4.6 subgroup moderated effects analysis

To explore possible reasons for heterogeneity, this paper presents subgroup analyses of highly heterogeneous outcome indicators. The intervention effect of mind-body exercise on patients with metabolic syndrome may be affected by different exercise forms, exercise intensity, exercise cycle, exercise frequency, exercise time, and other factors. However, these factors were not analyzed in subgroups because exercise intensity and exercise time were similar among the studies.

As shown in **Table 2**, exercise intervention programs may contribute to heterogeneity. Concerning exercise form, fitness qigong exhibited significant differences in all outcome indicators, yoga in waist circumference, and tai chi in fasting blood glucose, compared with controls. Regarding the exercise period, 10-12 weeks showed significant differences in systolic blood pressure, triglycerides, and HDL cholesterol, while 24-48 weeks exhibited significant differences in all outcome indicators compared with controls. In terms of exercise frequency, there was a significant difference in systolic blood pressure at 2-3 times/week, in BMI at 4-5 times/week, and in all outcome indicators at 6-7 times/week compared to the control group.

**Table 2:** Results of subgroup moderated effects.

Outcome indicators	Variables	Subgroup	N	MD/SMD	95%CI	*P*	I²/%	*P* ^PHeterogeneity^
Waist circumference	Exercise forms	yoga	341/354	-1.2	-2.02, -0.38	0.004	11%	0.35
		Tai chi	36/34	-1.03	-11.07, 9.01	0.84	0%	0.7
		fitness qigong	174/166	-4.11	-5.75, -2.47	<0.00001	40%	0.17
	Exercise cycle	10-12 weeks	65/66	-0.95	-2.64, 0.74	0.27	0%	0.9
		24-48 weeks	486/488	-2.54	-3.94, -1.15	0.0003	72%	0.0007
	Exercise frequency	2-3 times/week	185/216	-1.68	-3.44, 0.07	0.06	53%	0.12
		4-5 times/week	192/172	-0.87	-1.84, 0.11	0.08	0%	0.98
		6-7 times/week	174/166	-4.11	-5.75, -2.47	<0.00001	40%	0.17
Body mass index	Exercise frequency	4-5 times/week	60/49	-0.61	-0.87, -0.34	<0.00001	0%	0.72
		6-7 times/week	339/331	-1.72	-2.30, -1.14	<0.00001	55%	0.02
Systolic blood pressure	Exercise forms	yoga	341/354	-2.06	-5.42, 1.30	0.23	60%	0.03
		Tai chi	66/54	-2.23	-10.62, 6.15	0.6	66%	0.09
		fitness qigong	284/276	-4.59	-5.81, -3.37	<0.00001	0%	0.46
	Exercise cycle	10-12 weeks	92/94	-7.86	-11.86, -3.86	0.0001	0%	0.95
		24-48 weeks	599/590	-2.97	-4.97, -0.98	0.004	78%	<0.00001
	Exercise frequency	2-3 times/week	185/216	-4.4	-7.53, -1.27	0.006	0%	0.95
		4-5 times/week	222/192	0.25	-2.28, 2.78	0.85	51%	0.09
		6-7 times/week	284/276	-4.59	-5.81, -3.37	<0.00001	0%	0.46
Fasting blood glucose	Exercise forms	yoga	341/354	-0.11	-0.33, 0.12	0.34	49%	0.08
		Tai chi	66/54	0.49	0.13, 0.86	0.008	0%	0.67
		fitness qigong	429/421	-0.97	-1.63, -0.31	0.004	94%	<0.00001
	Exercise cycle	10-12 weeks	92/94	-1.08	-3.37, 1.20	0.35	97%	<0.00001
		24-48 weeks	744/735	-0.46	-0.85, -0.07	0.02	92%	<0.00001
	Exercise frequency	2-3 times/week	185/216	0.01	-0.18, 0.21	0.9	0%	0.59
		4-5 times/week	267/237	-0.01	-0.32, 0.30	0.94	62%	0.01
		6-7 times/week	384/376	-1.19	-1.98, -0.41	0.003	95%	<0.00001
Triglycerides	Exercise forms	yoga	341/354	0.07	-0.08, 0.22	0.34	0%	0.88
		fitness qigong	347/343	-0.5	-0.72, -0.28	<0.00001	41%	0.07
	Exercise cycle	10-12 weeks	92/94	-0.38	-0.75, -0.01	0.04	31%	0.23
		24-48 weeks	614/620	-0.25	-0.46, -0.04	0.02	65%	0.0004
	Exercise frequency	2-3 times/week	185/216	0.04	-0.15, 0.24	0.67	0%	0.85
		4-5 times/week	219/200	-0.02	-0.22, 0.18	0.86	4%	0.39
		6-7 times/week	302/298	-0.54	-0.82, -0.26	0.0001	57%	0.02
High-density lipoprotein cholesterol	Exercise forms	yoga	341/354	0.01	-0.14, 0.16	0.9	0%	0.56
		Tai chi	84/71	0.38	-0.26, 1.01	0.24	71%	0.03
		fitness qigong	277/273	1.05	0.76, 1.34	<0.00001	53%	0.02
	Exercise cycle	10-12 weeks	85/86	0.52	0.04, 1.00	0.03	58%	0.05
		24-48 weeks	617/612	0.60	0.25, 0.95	0.0007	88%	<0.00001
	Exercise frequency	2-3 times/week	185/216	0.04	-0.16, 0.23	0.7	0%	0.48
		4-5 times/week	285/254	0.26	-0.02, 0.55	0.07	58%	0.02
		6-7 times/week	232/228	1.17	0.83, 1.52	<0.00001	57%	0.03

#### 3.4.7 sensitivity analysis

To assess the stability of the meta-analysis results, sensitivity tests were conducted for all outcome indicators. The results showed that, by systematically excluding individual studies and reanalyzing, the point estimates of the combined effect size remained within the original effect size’s 95% confidence interval. Therefore, the results of the meta-analysis for each outcome indicator appear to be stable.

#### 3.4.8 Publication bias test

In this study, the insulin resistance index included fewer than 10 studies, and the test efficacy was insufficient for a publication bias test. Therefore, only the other outcome indexes were tested for publication bias. The results of the Begg test (**Table 3**) showed that P > 0.05, suggesting that there was no publication bias in the study.

**Table 3:** Results of Begg’s test.

Outcome Indicators	Z	*P*>|z|
Waist circumference	0.48	0.631
Body mass index	1.09	0.276
Systolic blood pressure	0.70	0.484
Diastolic blood pressure	0.99	0.322
Fasting blood glucose	1.60	0.110
Triglycerides	1.59	0.112
High-density lipoprotein cholesterol	1.64	0.100

## 4 Discussion

The results of this study demonstrate that mind-body exercise can reduce insulin resistance and obesity in patients with MetS and regulate blood pressure, fasting blood glucose, and lipid levels. These findings partially align with previous research (35). Existing studies have indicated the potential clinical effectiveness of mind-body exercise in improving MetS risk factors (35). Yoga has been shown to reduce waist circumference and systolic blood pressure in MetS patients (19). Tai Chi has antioxidative and blood glucose-lowering effects in older individuals with MetS (25). Additionally, fitness qigong effectively improves waist circumference, blood pressure, diastolic blood pressure, triglycerides, and HDL-C levels in MetS patients (17, 36). Furthermore, the impact of mind-body exercise on metabolic risk factors has been confirmed in other disease populations as well (37, 38).

This study reveals that the intervention effects of mind-body exercise on MetS exhibit dose-dependent and selective influences. It is recommended to carefully select exercise intervention plans for different outcome indicators. For improving insulin resistance, waist circumference, BMI, fasting blood glucose, and HDL-C, the preferred intervention is fitness qigong, 6-7 times per week, for 24-48 weeks. For improving SBP and TG, the preferred intervention is fitness qigong, 6-7 times per week, for 10-12 weeks. Mind-body exercise emphasizes the combination of consciousness and spirit, focusing on conscious control of body activities, achieving mind-body unity (39). Research suggests that mind-body exercise can reduce inflammation, alleviate oxidative stress, lower circulating free fatty acids, thereby improving insulin resistance and obesity. Additionally, mind-body exercise enhances glucose utilization, lowers blood glucose levels (17, 29, 40, 41), induces the synthesis of APOA1-carrying lipoproteins, and increases HDL-C levels (17, 33). On the neurological level, mind-body exercise can alter markers of sympathetic and parasympathetic nervous system activity (42), increase sensitivity to stress reflexes, decrease arterial tension and peripheral resistance, thereby improving blood pressure levels (43). Numerous studies indicate that fitness qigong exercise can improve MetS risk factors and reduce the risk of cardiovascular disease in MetS patients (16, 17, 28, 34, 44–47). Long-term regular mind-body exercise can improve MetS risk factors, and meta-analysis (17, 36) also supports the results of this study. Fitness qigong interventions for longer durations (≥6 months) have a better effect on improving BMI, HDL-C, and cholesterol, and 12-24 weeks of wuqinxi intervention can significantly reduce blood pressure.

This study also found that yoga effectively reduces waist circumference, tai chi effectively lowers fasting blood glucose, and fitness qigong effectively improves various risk factors, which is inconsistent with previous results. Existing meta-analyses have found that in healthy populations and those with a history and risk of cardiovascular disease or MetS, yoga can improve obesity, blood pressure, lipid abnormalities, glycated hemoglobin, and insulin resistance, but not fasting blood glucose (14, 48). Tai Chi can improve obesity, blood pressure, blood glucose, and lipid abnormalities (15). In MetS populations, yoga can improve waist circumference and systolic blood pressure (19), and fitness qigong can improve waist circumference, blood pressure, and lipid abnormalities (17), with no significant impact on fasting blood glucose. The different effects of various mind-body exercises on MetS risk factors may be due to differences in the types of studies included. This study only included randomized controlled trials (RCTs), while studies by Cramer (2014, 2016) included RCTs, randomized crossover trials, and cluster randomized trials. Differences in the health status of the study participants may also directly influence the different results, as this study only included MetS patients, while some studies included healthy individuals, those at risk of MetS, and MetS patients. The variations in the health status of the study participants could lead to different outcomes.

In summary, mind-body exercise can effectively improve risk factors such as insulin resistance in patients with MetS. The current evidence recommends the adoption of moderate-intensity fitness qigong, with an intervention frequency of 6-7 times per week for 24-48 weeks for MetS patients. This study suggests that the above exercise regimen could be promoted in populations at risk of MetS to prevent the occurrence and progression of metabolic syndrome. It can also serve as a basis for clinical guidance on exercise for individuals with MetS, providing tailored exercise prescriptions for improving various symptoms in middle-aged and older individuals with MetS. However, this study has several limitations: the literature search was conducted in both Chinese and English, potentially leading to incomplete literature inclusion. Additionally, the age, condition, and duration of MetS in the patients were not specified, and the intervention effects of different mind-body exercises may be influenced by age, condition, and disease duration.

## 5 Conclusion

Mind-body exercise is effective in improving risk factors in patients with metabolic syndrome. Current evidence recommends an intervention program of low to moderate intensity fitness qigong, with 6-7 sessions per week for 24-48 weeks in patients with metabolic syndrome.

## Data availability statement

The original contributions presented in the study are included in the article/supplementary material. Further inquiries can be directed to the corresponding author.

## Author contributions

SL: Conceptualization, Data curation, Writing – original draft, Methodology. PW: Data curation, Methodology, Writing – original draft. JW: Data curation, Writing – original draft. JZ: Data curation, Writing – original draft. XW: Data curation, Writing – original draft. TL: Writing – review & editing.
